# Application of the end-of-life demands card game and mindfulness-based cancer recovery program for reducing negative emotion in patients with advanced lung cancer: a randomized controlled trial

**DOI:** 10.3389/fpsyg.2025.1476207

**Published:** 2025-03-19

**Authors:** Xian Luo, Xiaoju Miao, Nana Ding, Zhongmin Fu, Xiaowen Wang, Yonghong Li

**Affiliations:** ^1^Department of Nursing, Affiliated Hospital of Zunyi Medical University, Zunyi, China; ^2^Department of Neurosurgery, Affiliated Hospital of Zunyi Medical University, Zunyi, China; ^3^The Affiliated Hospital of Guizhou Medical University, Guiyang, Guizhou, China; ^4^The First People’s Hospital of Zunyi, Zunyi, Guizhou, China

**Keywords:** card game, mindfulness-based cancer recovery, advanced lung cancer, death anxiety, psycho-oncology, psychosocial intervention

## Abstract

**Background:**

Lung cancer is the leading cause of cancer-related deaths globally and the most common type of cancer in China, posing significant health and socio-economic challenges. Despite the effectiveness of psychological interventions for death anxiety, discussions around death are often avoided in China due to cultural taboos and neglect of end-of-life care.

**Purpose:**

This study aimed to explore the effectiveness of the self-designed end-of-life demands card game (ELDCG) and mindfulness-based cancer recovery program (MBCR) programs in alleviating death anxiety, anxiety, depression, and stress in patients with advanced lung cancer.

**Methods:**

This was a randomized, single anonymized study. We randomly assigned 77 patients into two groups: the intervention group (38 patients) and the control group (39 patients). Routine health promotion was implemented in the intervention group, along with the ELDCG combined with the MBCR program, while the control group only received routine health promotion. The intervention lasted for 6 weeks. The intervention group completed the ELDCG within the first week, and the MBCR was completed during the remaining 5 weeks. The primary outcome was the Templer’s Death Anxiety Scale (T-DAS) score, and the secondary outcomes were the Hospital Anxiety and Depression Scale (HADS) score, the score on the Chinese version of the Perceived Stress Scale (CPSS), and frequency of selection in the ELDCG. The assessment results of these scales were collected both before the intervention began and after the 6-week intervention period. The frequency of card selections was recorded after the ELDCG.

**Results:**

After implementing the 6-week ELDCG and MBCR program, the intervention group showed significantly lower scores than the control group in death anxiety (*p* < 0.001), anxiety (*p* < 0.001), depression (*p* < 0.001), and stress (*p* < 0.001). The card with the highest selection frequency (13 times) was “I need the right to choose treatment options and understand the expected outcomes.”

**Conclusion:**

The ELDCG might assist patients in emotionally coming to terms with death, while the MBCR offered potential strategies for managing stress. Together, they seemed to alleviate death anxiety and negative emotions by addressing these psychological factors, which in turn improves patients’ ability to manage their illness. This improvement not only enhances their quality of life but also helps prevent the unnecessary consumption of healthcare resources, thus alleviating some of the financial strain on the healthcare system. Future research should assess the long-term effects and explore broader applications for terminally ill patients.

**Clinical trial registration:**

https://clinicaltrials.gov/, identifier ChiCTR2400081628.

## 1 Introduction

Lung cancer is the primary cause of cancer-related deaths worldwide and the second most prevalent cancer (Siegel et al., 2021). It severely threatens human health and creates a substantial socio-economic burden. In China, lung cancer has the highest incidence and mortality rates among malignancies, presenting a significant societal challenge (Cao et al., 2021). Patients with lung cancer, particularly in advanced stages, often experience elevated levels of negative emotions, like death anxiety, depression, and stress (Naser et al., 2021; Hong et al., 2022; Wang et al., 2021). Higher levels of these negative emotions can severely impact patients’ physical and mental health or even increase morbidity and mortality rates (Antoniadis et al., 2023; Chien et al., 2018). Thus, they can significantly reduce patient’s quality of life and result in an overuse of healthcare resources.

Several studies reported that psychological interventions effectively reduced negative emotions such as anxiety, depression, and stress (Sheard and Maguire, 1999; Pitceathly et al., 2009). However, in clinical practice, discussions on death and death anxiety are rare. These topics are not typically included in psychotherapist training, which may result in therapists failing to recognize potential death anxiety in patients (van Dam, 2016). Adverse psychological outcomes in cancer patients, including depression, dependency, and fear of pain and dying, largely stemmed from death anxiety (Lo et al., 2010), single psychological interventions were often insufficient to address this death anxiety (Lu et al., 2024). Death education was to help individuals understand and view death healthily and positively (Veatch, 1982). Although some studies explored various approaches to death education, few patients were willing to engage in such programs (Kim Y. H. et al., 2016; Kim B. R. et al., 2016; Kawagoe and Kawagoe, 2000). Most people tended to avoid or fear discussing death. Social and cultural taboos surrounding death made it difficult for patients to confront these issues openly.

To encourage more natural conversations about death, the Coda Alliance in the United States developed 36 cards that individuals can select from Osman et al. (2018), then record and understand their primary and most important needs according to the cards selected. Delgado-Guay et al. (2016) conducted a randomized controlled trial to guide patients in facing death by choosing the “Go Wish” cards and sharing the reasons behind their choices, which helped alleviate death-related stress without exacerbating anxiety. The card-based game can be used to provide education about death and as a tool for understanding patients’ end-of-life needs. The Chinese American Coalition for Compassionate Care (CACCC) developed heart-to-heart Cards based on the “Go Wish” cards from the Coda Alliance, incorporating elements of poker and practical experience (Jia et al., 2021). The wish cards were designed in Chinese and intended to discuss end-of-life topics. It was suitable for hosting tea parties at any stage of life, where participants could converse about life and death while enjoying tea (Duan et al., 2024). These discussions aimed to enhance participants’ understanding of death, make the most of their remaining time, and address personal preferences for medical care, considerations about the quality of life, and arrangements for the afterlife. However, considering these cards originate from European countries, some of their content may not be fully applicable to the Chinese cultural context, such as: “Do not touch the body within a few hours after death,” “I want my religious relics by my bedside,” and “Do not place the body in a morgue within 8 h after death” (Jia et al., 2021). The reason lies in the differences between Chinese religious culture and funeral customs and those of Western countries. We developed the End-of-Life Demand Card Game (ELDCG), based on Go Wish cards, which was culturally adapted to explore death education methods for patients within the Chinese context.

Additionally, mindfulness levels were related to death anxiety. Higher levels of mindfulness were associated with a more positive attitude toward death (Cacciatore et al., 2015). The Mindfulness-Based Cancer Recovery (MBCR) program is based on mindfulness-based stress reduction and incorporates knowledge from the fields of cancer research and psychology (Chayadi et al., 2022). It was reported that the 6-week MBCR program could alleviate anxiety, depression, cancer-related pain, sleep disorders, and other conditions in cancer patients (Chayadi et al., 2022). Speca et al. (2000) randomly assigned 89 patients with malignant tumors into mindfulness-based intervention and control groups. The results showed that 35% of patients in the mindfulness-based intervention group experienced reduced stress symptoms, and 65% showed improved emotional fluctuations. Carlson et al. (2016) randomly allocated 252 stage I-III breast cancer patients into MBCR and Supportive-Expressive Therapy groups. The findings indicated that compared to the supportive-expressive therapy group, the MBCR group effectively reduced emotional disturbances (primarily fatigue, anxiety, and confusion) and stress symptoms (including tension, sympathetic arousal, and cognitive symptoms). Rohánszky et al. (2017) studied the effectiveness of MBCR in 101 cancer patients and assessed them before intervention (T1), at 8 weeks post-intervention (T2), and 6 months post-intervention (T3). The results showed a significant improvement in the quality of life in the MBCR group between T1 and T2, with decreased levels of stress and depression and increased levels of optimism and vitality. These positive changes persisted at the end of the follow-up period (T3).

Based on previous literature, we have found that mindfulness interventions and card games can both have positive effects on cancer patients. However, there is currently a lack of effective and scalable intervention programs, which leaves death education and intervention for negative emotions and stress among cancer patients in a rather critical state. To address this issue, this study proposes an integrated intervention program that combines mindfulness interventions with card games. We integrated ELDCG with MBCR to offer both emotional support and practical psychological adjustment, enabling patients to address the end of life more comprehensively and profoundly. Therefore, the purpose of this study was to explore the effectiveness of combining mindfulness-based cancer recovery (MBCR) with an end-of-life demands card game (ELDCG) in reducing death anxiety, negative emotions, and stress in cancer patients.

## 2 Methods

### 2.1 Study design

We conducted a parallel, data analyst-anonymized, two-arm, randomized controlled study in which the ELDCG and MBCR programs were applied in patients with advanced lung cancer.

This study was conducted in the oncology ward of Zunyi Medical University’s Affiliated Hospital from May to November 2021. Recruitment began when patients were admitted, with each being informed about the study’s aims and importance before invitation to participate. Those who agreed gave their informed consent. Out of 108 initially enrolled patients, 84 met the inclusion criteria. These patients were selected as study subjects by random sampling. The inclusion criteria for this study were as follows: (1) ≥18 years of age and voluntary participation; (2) stable vital signs and awareness of the diagnosis; (3) clear pathological or cytological diagnosis of stage III or IV lung cancer; (4) a sufficient level of comprehension, at least at a primary school education level; (5) willingness to discuss death-related topics; (6) The physician (associate chief physician or higher) estimates that the hospitalization will last more than a week. Patients diagnosed with mental disorders or severe audiovisual disturbances or who were not cooperative were excluded.

### 2.2 Sample size

Before the start of the trial, we conducted a pilot study with 20 patients. Based on pilot study results, the sample size required to achieve an anticipated effect size of a mean difference in total Templer’s Death Anxiety Scale (T-DAS) score of 3.1 (with an SD of change 4.4) was calculated to be 33 patients in each group (an 80% power and a 5% error). Considering a dropout rate of 20%, we estimated that approximately 84 patients would be required (42 per group).

### 2.3 Randomization and anonymized

Before the trial, the research assistant explained the study’s purpose to patients and families, ensuring informed consent was obtained and documented. Baseline data were recorded upon inclusion and before randomization. Participants were then randomly assigned at a 1:1 ratio to the ELDCG and MBCR program (intervention) group or the routine health promotion group (control). Randomization was performed by using a computer-generated table of random numbers. The grouping information was printed inside opaque envelopes and numbered from 1 to 84 on the surface of the envelopes. After the envelopes were sealed, they were handed over to the researchers. When a patient who met the inclusion criteria entered the study, the researcher assigned a code and then opened the corresponding envelope. The allocation sequence for randomization was created before the study initiation by an independent administrator.

The study was single-anonymized since participants were anonymized for randomization and treatment. As both ELDCG and MBCR are highly personalized and interactive interventions, the intervention facilitators must engage in close interaction and guidance with the patients during the process, making implementers blinding unfeasible. The independent study statistician was anonymized for endpoint assessment.

### 2.4 Intervention methods

**Figure 1:**
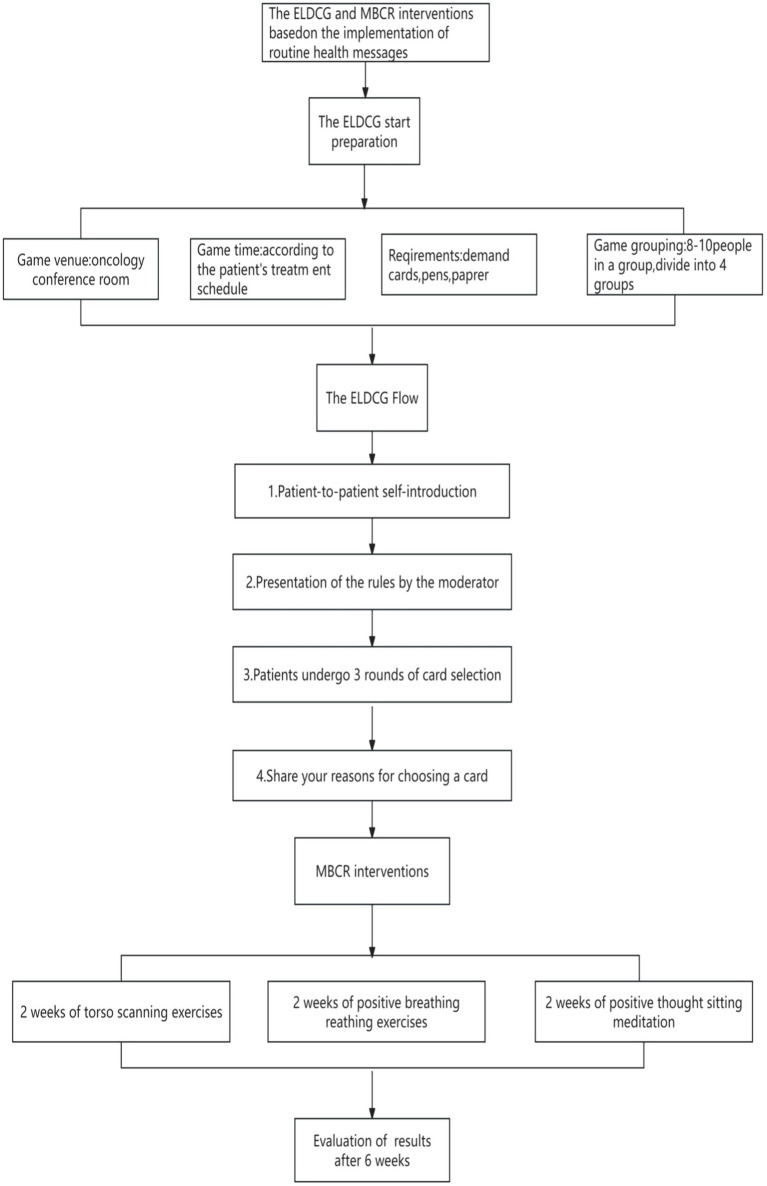
The ELDCG and MBCR program intervention flowchart.

The intervention methods were developed through a literature review and expert consultation, with two rounds of revisions. As shown in Figure 1, the intervention group participated in the ELDCG and a 6-week MBCR program, along with routine health promotion. In the first week, the ELDCG began 1–3 days after enrollment. Patients were divided into four groups of 8–10, with one group per round. Family members could accompany participants. A game-recording staff member and a psychological counselor facilitated the session. Patients selected cards and shared reasons for their choices. The MBCR was conducted the day after the ELDCG, with three training sessions, including 2 weeks of torso scanning exercises, 2 weeks of positive breathing exercises, and 2 weeks of positive thought sitting meditation. The first session was face-to-face, and the second and third were via telephone. The control group received only routine health promotion. Both groups underwent the same measurements before and after the intervention, while the intervention facilitators received training on ELDCG and MBCR through WeChat notifications or in-person lectures before patient enrollment. The process of developing the demand cards and the specific interventions applied to the two groups are detailed in Appendices 1, 2, respectively.

### 2.5 Outcomes

The primary outcome was the score on the T-DAS (Sharif Nia et al., 2021). The secondary outcomes included the Hospital Anxiety and Depression Scale (HADS) (Annunziata et al., 2020) and the Chinese version of the Perceived Stress Scale (CPSS) (Meng et al., 2020). The assessment results of these scales were collected both before the intervention began and after the 6-week intervention period.

We also assessed the frequency of card selection in the ELDCG.

*T-DAS* (Sharif Nia et al., 2021): We used the Chinese version of the scale, which consists of 15 items, covering four dimensions: cognition, emotion, time awareness, and stress and pain (Yang et al., 2013). The test–retest reliability of the scale is 0.83, and the internal consistency Cronbach’s *α* coefficient is 0.73. The total score ranges from 0 to 15, with a score ≥ 7 indicating high death anxiety and < 7 indicating low death anxiety.

*HADS* (Annunziata et al., 2020): The Cronbach’s α coefficient for the anxiety subscale is 0.92, and that for the depression subscale is 0.84. The Cronbach’s α coefficient for the anxiety subscale is 0.92. The HADS consists of two parts: the Anxiety subscale (HADS-A) and the Depression subscale (HADS-D), each with seven items. A score greater than seven on the anxiety or depression dimension suggests the possible presence of anxiety or depression.

*CPSS* (Meng et al., 2020): The Cronbach’s α coefficient is 0.78. The scale uses a 5-point scoring system and consists of 14 items reflecting feelings of stress and loss of control. The total score ranges from 14 to 70, with scores of 43–56 indicating high stress and scores of 57–70 denoting very high stress.

We also collected the distribution of the top 10 ELDCG selections for advanced lung cancer patients by frequency in the intervention group.

### 2.6 Ethics statement

The study was approved by the Ethics Committee of Zunyi Medical University Affiliated Hospital (Approval No: KLLY-2020-144), registered with the Chinese Clinical Trials Registry (ChiCTR2400081628). All patients enrolled in the study voluntarily.

### 2.7 Statistical methods

After the data collection, they were entered into Excel 2016 and analyzed using SPSS 18.0. The frequencies and percentages are used to describe categorical data. For normally distributed continuous data, the mean and standard deviation (mean ± SD) is used. For the score on T-DAS, HADS, and CPSS, an independent sample *t*-test was used for between-group comparisons, and a paired sample *t*-test was used for before-after comparisons. For sociodemographic data, the chi-square (χ^2^) test was used. The significance level was set at *α* = 0.05, with a *p*-value <0.05 considered to indicate statistical significance.

## 3 Results

### 3.1 Sociodemographic data

**Figure 2:**
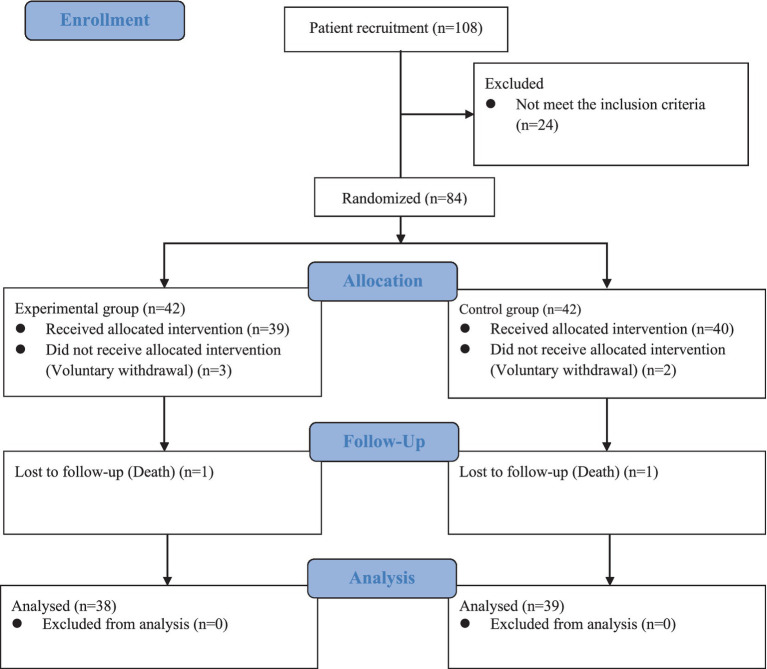
Flow diagram of this study.

A total of 108 patients were enrolled in our study, of whom 84 met the inclusion criteria. Seven patients were excluded due to voluntary withdrawal (*n* = 5) or death (*n* = 2). The final sample comprised 77 patients (intervention group = 38, control group = 39; Figure 2). The detailed sociodemographic data can be found in Table 1. No significant difference was found in the sociodemographic data between the two patient groups before the intervention (*p* > 0.05). Among the patients, 41.56% were aged 18–44, and 29.87% were aged 49–59. Most had low education levels, with 32.47% having primary education and 48.04% having junior high school education.

**Table 1:** Comparison of sociodemographic data between the two groups of advanced lung cancer patients [*n* (%)].

Project	Intervention group (*n* = 38)	Control group (*n* = 39)	Total (*n* = 77)	*χ* ^2^	*p*
Age					
18–44	16 (42.10)	16 (41.02)	32 (41.56)	0.03	0.99
45–59	11 (28.95)	12 (30.77)	23 (29.87)		
60–80	11 (28.95)	11 (28.21)	22 (28.57)		
Education level					
Primary	15 (39.47)	10 (25.64)	25 (32.47)	−1.61	0.11
Junior School	18 (47.37)	19 (48.72)	37 (48.04)		
High School	4 (10.52)	7 (17.95)	11 (14.29)		
Junior College	0 (0.00)	3 (7.69)	3 (3.90)		
College	1 (2.63)	0 (0.00)	1 (1.30)		
Income					
Below 1,000¥	9 (23.68)	9 (23.08)	18 (23.38)	0.70	0.95
1,000–2,999¥	10 (26.32)	10 (25.64)	20 (25.98)		
3,000–4,999¥	7 (18.42)	10 (25.64)	17 (22.08)		
5,000–9,999¥	6 (15.79)	5 (12.82)	11 (14.29)		
10,000 and above	6 (15.79)	5 (12.82)	11 (14.29)		
Marital status					
Married	36 (94.74)	36 (92.31)	72 (93.51)	0.32	0.85
Divorced	1 (2.63)	1 (2.56)	2 (2.60)		
Widowed	1 (2.63)	2 (5.13)	3 (3.89)		
Religion					
None	36 (94.74)	38 (97.44)	74 (96.10)	0.37	0.54
Yes	2 (5.26)	1 (2.56)	3 (3.90)		

### 3.2 Effect of the ELDCG combined with the MBCR program on reducing death anxiety in lung cancer patients

Before the intervention, no significant differences in the total (*p* = 0.77) or each dimension (*p* > 0.05) scores for death anxiety were observed between the two groups. After the intervention for 6 weeks, the total and dimension scores for death anxiety were lower in the intervention group than in the control group (*p* < 0.05). In addition, the total and each dimensional death anxiety score in the intervention group were lower after the intervention than before the intervention (*p* < 0.05) (Table 2).

**Table 2:** Comparison of death anxiety scores between the two groups.

Dimension	Group	Before intervention (Mean ± SD)	After 6-weeks intervention (Mean ± SD)
Emotion	Control (*n* = 39)	2.10 ± 1.25	2.13 ± 1.32
	Intervention (*n* = 38)	2.05 ± 1.11	0.79 ± 0.34^▲^
	*t*	−0.19	−5.20
	*p*	0.85	<0.001
Stress and pain	Control (*n* = 39)	1.82 ± 0.73	1.23 ± 0.87
	Intervention (*n* = 38)	1.54 ± 0.55	0.61 ± 0.34^▲^
	*t*	1.88	−3.07
	*p*	0.06	0.003
Time awareness	Control (*n* = 39)	1.08 ± 0.66	1.05 ± 0.65
	Intervention (*n* = 38)	0.97 ± 0.25	0.63 ± 0.36 ^▲^
	*t*	−0.64	−2.56
	*p*	0.53	0.013
Cognitive	Control (*n* = 39)	1.87 ± 0.99	1.74 ± 0.68
	Intervention (*n* = 38)	1.85 ± 0.96	1.13 ± 0.56^▲^
	*t*	0.11	−2.60
	*p*	0.92	0.01
Total score	Control (*n* = 39)	6.71 ± 2.08	6.15 ± 2.36
	Intervention (*n* = 38)	6.56 ± 2.28	3.16 ± 2.21^▲^
	*t*	0.29	−5.75
	*p*	0.77	<0.001

▲ indicates *p* < 0.05 compared with before intervention group.

### 3.3 Effect of the ELDCG combined with the MBCR program on reducing anxiety and depression in lung cancer patients

Before the intervention, no significant differences in the anxiety (*p* = 0.32) or depression (*p* = 0.44) scores were found between the two groups. After receiving the intervention for 6 weeks, the anxiety or depression scores were lower in the intervention group than in the control group (*p* < 0.001). In addition, the anxiety or depression scores in the intervention group were lower after the intervention than before the intervention (*p* < 0.001) (Table 3).

**Table 3:** Comparison of hospital anxiety and depression scale scores between the two groups of patients.

Item	Group	Before intervention (Mean ± SD)	After 6-weeks intervention (Mean ± SD)
Anxiety dimension	Control (*n* = 39)	11.15 ± 2.82	11.39 ± 2.09
	Intervention (*n* = 38)	10.63 ± 1.57	5.87 ± 2.53^▲^
	*t*	−1.01	−10.46
	*p*	0.32	<0.001
Depression dimension	Control (*n* = 39)	12.31 ± 2.53	11.46 ± 3.14
	Intervention (*n* = 38)	12.68 ± 1.66	5.82 ± 3.22^▲^
	*t*	0.77	−7.78
	*p*	0.44	<0.001

▲ indicates *p* < 0.05 compared with before intervention group.

### 3.4 Effect of the ELDCG combined with the MBCR program on reducing stress in lung cancer patients

Before the intervention, no significant differences in the total (*p* = 0.32) or dimension (*p* > 0.05) scores for perceived stress were found between the two groups. After receiving the intervention for 6 weeks, the total and dimension scores for perceived stress were lower in the intervention group than in the control group (*p* < 0.001). In addition, the total and dimensional stress scores in the intervention group were lower after the intervention compared to before the intervention. (*p* < 0.001) (Table 4).

**Table 4:** Comparison of perceived stress scale scores between the two groups of patients.

Item	Group	Before intervention (Mean ± SD)	After intervention (Mean ± SD)
Tension dimension	Control (*n* = 39)	19.59 ± 3.48	19.56 ± 2.34
	Intervention (*n* = 38)	20.74 ± 1.22	14.47 ± 2.20^▲^
	*t*	1.94	−9.83
	*p*	0.06	<0.001
Loss of control dimension	Control (*n* = 39)	25.92 ± 3.62	25.36 ± 3.31
	Intervention (*n* = 38)	25.90 ± 1.66	21.29 ± 2.39^▲^
	*t*	−0.04	−6.19
	*p*	0.97	<0.001
Total perceived stress score	Control (*n* = 39)	45.51 ± 6.51	44.92 ± 4.61
	Intervention (*n* = 38)	46.63 ± 2.20	35.76 ± 3.21^▲^
	*t*	1.02	−10.14
	*p*	0.32	<0.001

▲ indicates *p* < 0.05 compared with before intervention group.

### 3.5 Selection of cards in the ELDCG

The game was conducted over four sessions, and the researchers recorded the selection of demand cards. The top 10 items, according to frequency, are listed in Table 5. Among these 10 items, “I need the right to choose treatment options and understand expected outcomes” was considered the most important demand.

**Table 5:** Distribution of the Top 10 ELDCG selections for advanced lung cancer patients by frequency.

Number	Item	Frequency
B5	I need the right to choose treatment options and understand the expected outcomes	13
A11	I need to understand the progression of my illness	12
E4	I need to minimize regrets in my final moments	11
C3	I do not want my family to show sadness in front of me	11
E1	I need to address unfinished matters with family and friends in reviewing my life	10
A2	I need doctors and nurses to have a comprehensive understanding of my condition	8
E6	I need close companionship when I am in pain	7
C6	There needs to be better communication between my family and me	6
D5	I need respect from society, not just sympathy and pity	6
E7	I need to express love, apologize, thank, and say goodbye to important people in my life	6

## 4 Discussion

This trial was the first to assess the efficacy of combining a demand card game with the MBCR program for cancer patients. We found that combining the demand card game, as a form of death education, with MBCR training effectively reduces death anxiety, depression, and stress in patients with advanced lung cancer.

Our study found that combining the ELDCG with the MBCR program effectively reduced death anxiety, anxiety, depression, and stress in patients. Tomer and Eliason (1996) suggested that long-term death-related stress can develop into chronic anxiety, significantly affecting mental and physical health. Many advanced lung cancer patients face death-related thoughts, which foster fear and anxiety, leading to a worse quality of life (Adorno and Wallace, 2017). If unresolved, these anxieties can result in negative emotions. Previous studies show that death education and mindfulness interventions can reduce death anxiety (Veatch, 1982; Cacciatore et al., 2015), but research combining both interventions is limited. The DACUM death education program, developed in Korea, improved the psychological well-being of cancer patients (Kim B. R. et al., 2016). Non-experiential death education often has negative impacts, while experiential death education shows rather positive effects (Lekes et al., 2022), potentially offering benefits in coping with death anxiety (Paiva et al., 2023), and Dupont et al. (2022) reported that the “Go Wish” cards are effective for discussing death with advanced cancer patients. Compared to previous card-based games (Jia et al., 2021), the ELDCG we designed was more culturally appropriate for Chinese patients, enabling them to explore death-related thoughts and concerns. However, ELDCG alone was insufficient to significantly reduce anxiety and stress. Thus, we incorporated MBCR to further address these issues. MBCR promoted mindfulness and emotional regulation, helping patients manage their fear of death and face illness with emotional balance (Pedro et al., 2021). Yu et al. (2019) showed that mindfulness reduced stress and enhanced emotional acceptance. The MBCR program, designed with psychological experts, was delivered both online and offline to adjust to varying hospitalization durations and maintain patient engagement. Patients who received ELDCG combined with MBCR were guided to actively cope with their illness, leading to a reduction in anxiety, depression, and stress.

In our study on card selection in the ELDCG among patients with advanced lung cancer, we found that their end-of-life demands focused primarily on how to live better, with a limited selection of items related to high-quality dying. These items included (i) drafting an advance directive while mentally competent, (ii) seeking family opinions before life support decisions, and (iii) stopping painful treatment when beyond cure. Studies show low awareness of advance care planning among the Chinese population (Kang et al., 2017; Ni et al., 2021), exacerbated by insufficient end-of-life care services and a lack of education for nursing staff on death, hindering effective end-of-life discussions. This hinders the integration of death education into clinical practice and impacts patients’ understanding of death (Mehnert and Koch, 2008). Additionally, the absence of relevant laws in China highlights the need for legal frameworks that support the implementation of death education for cancer patients. This issue is deeply tied to Chinese cultural and social context, where Confucianism influences attitudes toward death as a balance between valuing life and fearing its end (Lei, 2021). Taoism and Buddhism also impact these perspectives, with Taoism emphasizing longevity, challenging the acceptance of mortality (Bai and Yin, 2014). The ELDCG facilitates a relaxed environment for patients to express emotions without overwhelming psychological burdens, avoiding direct references to “death” in favor of terms like “farewell” or “passing” to reduce cultural and emotional barriers. The game also reveals patients’ subconscious emotions, as three patients cried during the game and had difficulty articulating their thoughts. Two female patients later expressed fear of not being able to care for their children as they grew up, reflecting the guilt mothers in Chinese society may feel about their inability to provide for their children at the end of life. The ELDCG provides emotional comfort by helping patients reframe their role as mothers, focusing on the emotional legacy they leave behind.

### 4.1 Clinical implications

This study suggests that combining the ELDCG with the MBCR program can effectively reduce death anxiety, depression, and stress in advanced lung cancer patients. This approach offers a promising, culturally tailored intervention that can be integrated into clinical practice to improve psychological well-being and to support end-of-life discussions. Ultimately, the combination of ELDCG and MBCR can enhance the quality of life for patients facing terminal illness.

### 4.2 Limitations

This study has several limitations. First, the ELDCG was only implemented once, making it challenging to separate its effects from those of the MBCR program. Additionally, there is a lack of comparative studies between ELDCG, MBCR, and their combination. Future research should focus on comparing the effects of these three approaches. Second, as a single-center study with a small sample size, the findings, though applicable to advanced lung cancer patients, may not be widely generalizable. Third, because the intervention providers were not anonymized, there is a possibility that their behavior may have unintentionally influenced the participants. Finally, since patients were in the advanced stages of cancer and had limited energy, to reduce their burden and improve intervention adherence, the 8-week MBCR was shortened to 6 weeks.

## 5 Conclusion

The combination of ELDCG and MBCR effectively addresses key psychological issues such as death anxiety, depression, and stress in patients with advanced lung cancer. These psychological burdens, if left unmanaged, can severely affect the patients’ quality of life, hinder their ability to cope with illness, and lead to the overuse of healthcare resources. By alleviating these psychological factors, the intervention not only improves the patient’s well-being but also helps optimize healthcare resource usage, thereby reducing the financial strain on the healthcare system.

## Data Availability

The raw data supporting the conclusions of this article will be made available by the authors, without undue reservation.
