# Supplementary material for: Application of the end-of-life demands card game and mindfulness-based cancer recovery program for reducing negative emotion in patients with advanced lung cancer: a randomized controlled trial
Source: Front Psychol. 2025 Mar 19;16:1476207. doi: 10.3389/fpsyg.2025.1476207 (PMC11961935; doi:10.3389/fpsyg.2025.1476207)
Supplement: Supplementary file 1 [file Table_1.docx]

Appendix 1

The process of selecting end-of-life demand cards

1. End-of-life demand cards content source: The content of the ELDCG was converted from the self-designed "End-of-life Needs Questionnaire for Patients with Advanced Cancer", which contains 50 items in 5 dimensions. The Cronbach's α coefficient of internal consistency was 0.935, and the Cronbach's α coefficient of each dimension was between 0.934 and 0.945. The test-retest reliability correlation coefficient was 0.859. The I-CVI is between 0.83 and 1, and the S-CVI/Ave is 0.95. Exploratory factor analysis revealed that the total cumulative explanatory variable was 65.514%.

2. Production of demand cards: The size of the card is 57*87 mm, and each deck is composed of 50 alternative cards and 2 blank supplementary cards. The back of the card is used to represent 5 different dimensions and supplementary cards with 6 colors, among which dark blue is the medical service demand dimension (12 cards), coded with the letters A1-A12; light blue represents the medical choice needs dimension (7 photos), coded with the letters B1-B7; the red represents the psychological needs dimension (11 photos), coded with the letters C1-C11; the dark green represents the social needs dimension (10 photos), coded with the letters D1-D10; and the yellow represents the spiritual needs dimension (10 photos), coded with the letters E1-E10. The other two supplementary cards are light green.


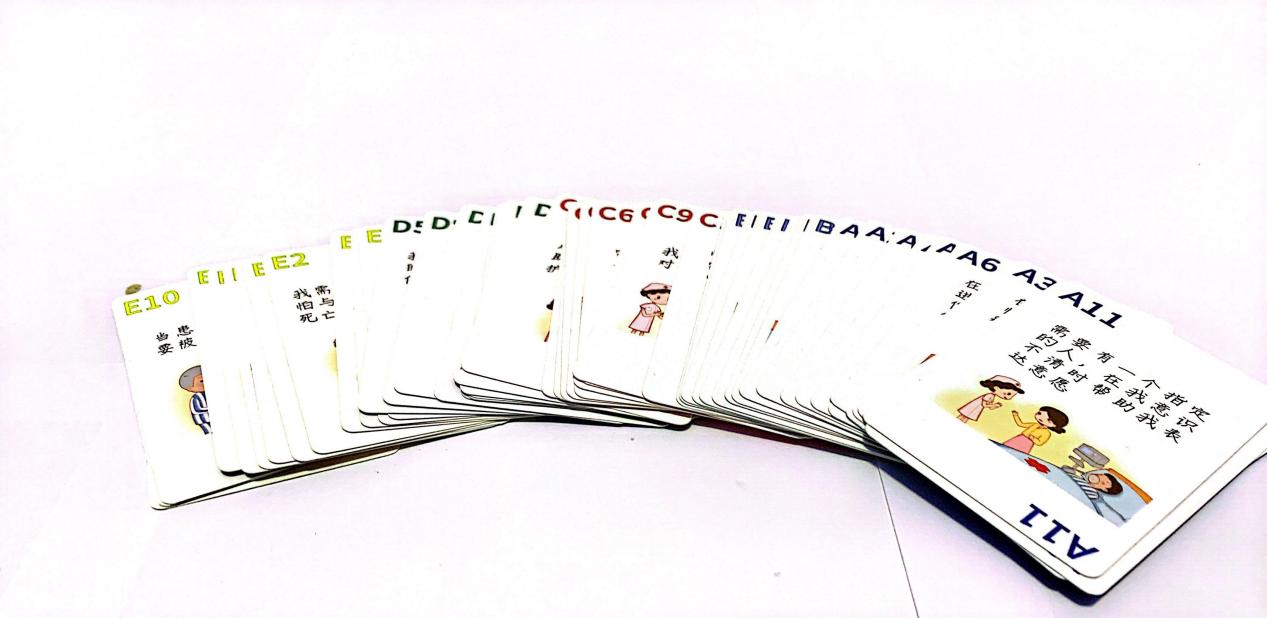


Figure 1. Demand card display


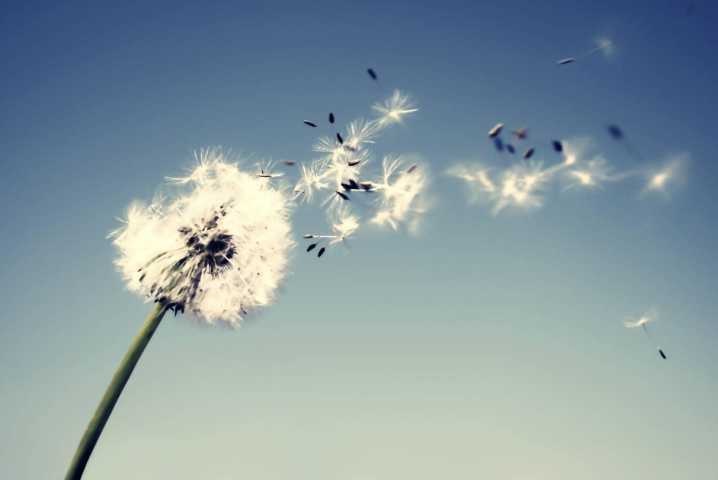

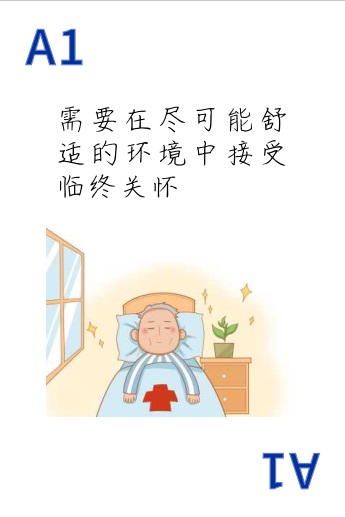


Figure 2 Both sides of a medical service demand card


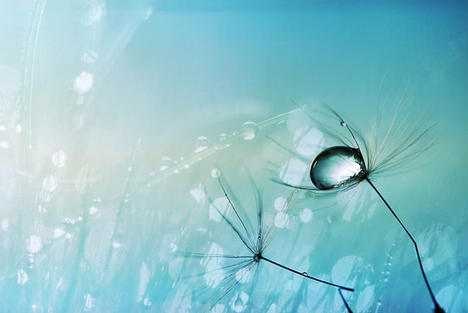

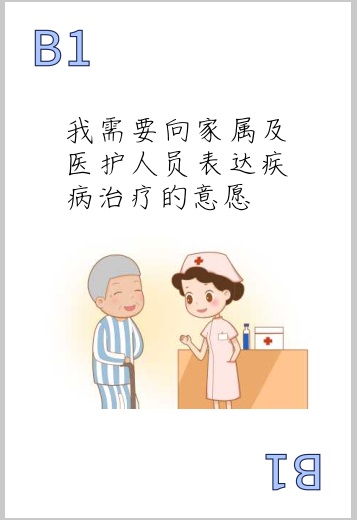


Figure 3 Both sides of a demand for medical choice card


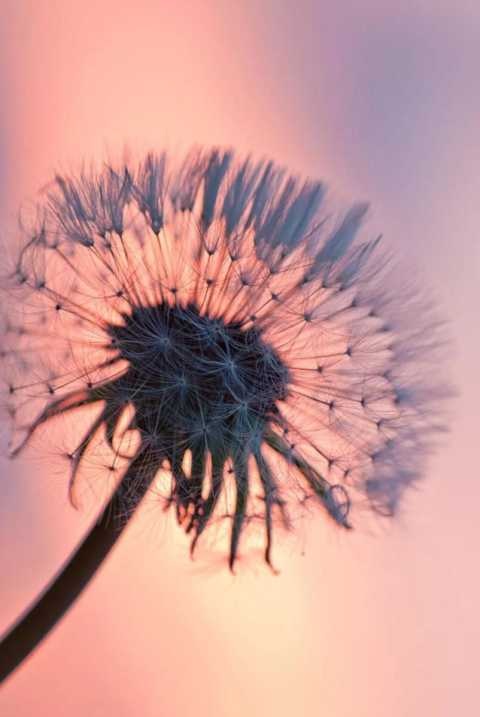

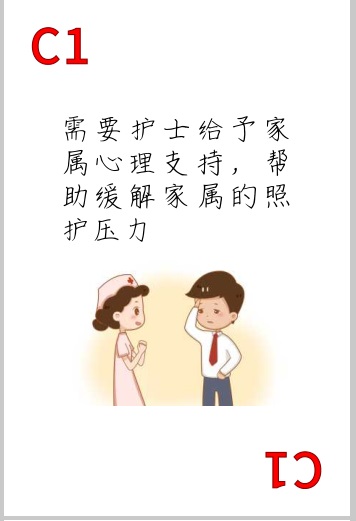


Figure 4 Both sides of a psychological needs card


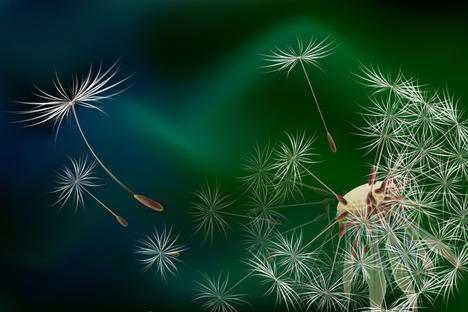

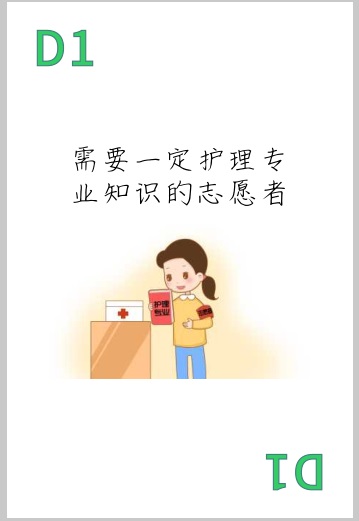


Figure 5 Both sides of a social needs card


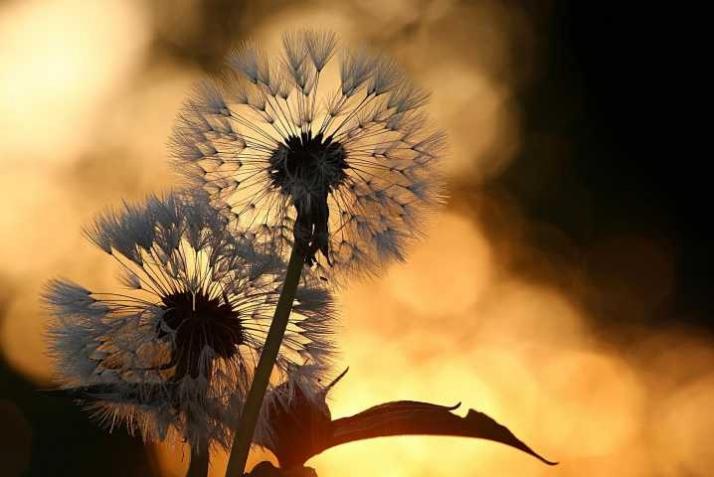

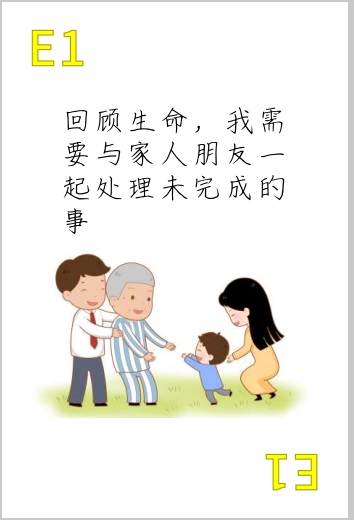


Figure 6 Both sides of a spiritual needs card


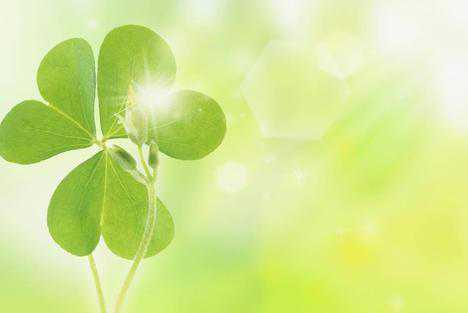

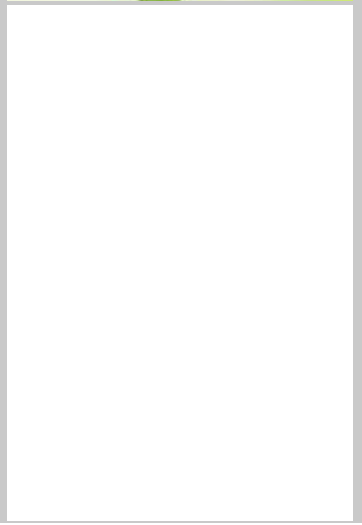


Figure 7 Both sides of a blank card
