# Supplementary material for: Application of the end-of-life demands card game and mindfulness-based cancer recovery program for reducing negative emotion in patients with advanced lung cancer: a randomized controlled trial
Source: Front Psychol. 2025 Mar 19;16:1476207. doi: 10.3389/fpsyg.2025.1476207 (PMC11961935; doi:10.3389/fpsyg.2025.1476207)
Supplement: Supplementary file 2 [file Table_2.docx]

Appendix 2

**1. Control group intervention**

The control group adopted the routine nursing education method. Researchers consulted the literature and evaluated limited knowledge related to cancer in combination with departmental brochures. Within six weeks, they pushed a piece of knowledge to patients every week through WeChat to answer questions raised by patients in a timely manner. See Table 1 for details.

| **Table 1 Content of health education in the control group** | |
| --- | --- |
| **project** | **content** |
| Dietary recommendations for cancer patients | Recommend some green anticancer foods to patients; introduce their efficacy and simple cooking methods. |
| Dietary precautions for cancer patients | Introduce cancer diet misunderstandings and dietary contraindications to patients. |
| abdominal respiration | Introduce the benefits and methods of abdominal breathing for lung cancer patients. |
| Treatment of adverse reactions to chemotherapy | Introduce common adverse reactions during chemotherapy to patients |
| Seven myths about analgesics | Myth 1: Cancer pain can endure, cannot help but take medicine; Myth 2: Early use of analgesics will not be available in the future; Myth 3: Pain treatment only provides partial relief; Myth 4: Take medicine when in pain, do not take medicine when not in pain; Myth 5: Common painkillers must be safer than opioids; Myth 6: Stopping the drug too soon can lead to withdrawal symptoms. |
| The influence of psychology on disease | Introduce the effects of a negative attitude and good attitude on the central nervous system, endocrine system and body immune system to patients and emphasize the importance of maintaining a good attitude. |

**Intervention group**

1. the ELDCG combined with the MBCR program

2.1 Preparation before the game: ① Game venue: Select the conference room of the oncology department as the game location, adjust the appropriate indoor temperature before the game starts, and place appropriate amounts of tea and cakes for patients to consume on the day of the game; ② Game time: Make a patient treatment schedule, choose the game time according to the schedule, and generally start at 15:00 in the afternoon to ensure that it does not affect patients’ treatment and ensure sufficient rest. The duration of each round is controlled within 60-90 min. ③ Material preparation: end-of-life demand cards, game feedback form, pen, paper, and PPT.

2.2 Participants: The intervention begins 1-3 days after patients are enrolled, and patients in the intervention group are divided into 4 groups of 8-10 people in order of enrollment. One group of patients per round of the end-of-life demands card game. Family members may accompany the participants. There is also one host, two game recording staff members, and one psychological counsellor.

2.3 Game process: ① The host first invites the patients to introduce themselves, and the host subsequently guides the patients to meditate on the last day of life as the theme; ② Introduce the game card content, card selection purpose and rules; (3) Assign each person a pair of end-of-life demand cards. The game is divided into 3 rounds. The first round includes 50 cards from which patients choose their own important 10 cards, the second round includes 10 cards from which patients select 5 cards, and the third round includes 5 cards from which patients choose a card that they think is the most important. Patients can choose to supplement the card instead of no content; ④ After card selection, the patient arranges the first 5 cards selected in turn and share the reasons for card selection one by one.

2.4 MBCR intervention program: requires the MBCR program the day after the end of the card game

2.4.1 Intervention Program: In this study, patients who did not reach 80% of the prescribed exercises were considered to have not completed the intervention and were treated as "exit". For specific measures, see Table 2.

|  | **Table 2 MBCR intervention program** | | | |  | |  |
| --- | --- | --- | --- | --- | --- | --- | --- |
| **course** | **project** | **concrete content** | **Intervention time** | **homework** | | **Intervention form** | |
| First training | Body scanning exercise | Lung cancer related knowledge introduction, mindfulness introduction, body scanning exercise | Training begins the day after the completion of the temporary demand card activity | The practice time is 2 weeks, once per day, and the family practice record form is filled out. | | Face-to-face group training | |
| Second training | Mindful breathing exercises | The patient is instructed to listen to the recording of mindfulness practice and perform mindful breathing exercises | The training begins at the 3rd week after enrollment | The practice time is 2 weeks, once per day, and the family practice record form is filled out. | | One-to-one telephone coaching | |
| Third training | Mindfulness meditation | Instruct the patient to listen to a recording of the mindfulness meditation practice | The training begins at the 5th week after enrollment | The practice time is 2 weeks, once per day, and the family practice record form is filled out. | | One-to-one telephone coaching | |
